# Implementation of a decentralized hepatitis C care pathway for people who use drugs in Dutch addiction care. Study protocol for the Hepatitis C: chain of addiction care (CAC) project

**DOI:** 10.1186/s13722-022-00350-1

**Published:** 2022-11-30

**Authors:** Daan W. Von den Hoff, Floor A. C. Berden, Joost P. H. Drenth, Arnt F. A. Schellekens, HepNed, NISPA

**Affiliations:** 1 Department of Gastroenterology and Hepatology, Radboud University Medical Center, Geert Grooteplein Zuid 10, 6525 GA Nijmegen, The Netherlands; 2 Department of Psychiatry, Cognition and Behaviour, Radboud University Medical Center, Netherlands and Nijmegen Institute for Scientist-Practitioners in Addiction and Donders Institute for Brain, Radboud University, Nijmegen, The Netherlands

## Abstract

**Background:**

People who use drugs (PWUD) are at high risk for hepatitis C virus (HCV) infection and its complications. Given the high prevalence rate of HCV in PWUD, the World Health Organization (WHO) emphasizes PWUD as a target population for HCV elimination. The introduction of pangenotypic direct acting antivirals (DAAs) greatly simplifies HCV treatment, which encourages integration of HCV treatment in primary care. Facilitating low threshold HCV care for PWUD by implementing decentralized models is crucial for HCV elimination.

**Aims:**

With this study we aim to (1) eliminate 90% of identified HCV infections in Dutch addiction care, using a decentralized PWUD-HCV care model, and (2) identify facilitators and barriers for successful implementation of the model using interviews.

**Methods:**

We will perform a multicenter mixed-method study on HCV treatment in addiction care. In a prospective observational study we will examine HCV-related outcomes in PWUD receiving HCV treatment as part of addiction care. The primary outcome is viral elimination, defined as percentage of identified HCV positive patients cured with DAAs. In parallel, we will perform a qualitative study to explore facilitators and barriers for implementation of fully decentralized HCV-PWUD care. We will interview addiction care professionals and board members about their experience with HCV-care as part of addiction care.

**Discussion:**

This study will show effectiveness of integration of HCV care within addiction care, and provide insight in facilitators and barriers to implement integrated HCV-addiction care. The results will provide recommendations for implementation and maintenance of the decentralized HCV pathway, which can facilitate scaling-up to contribute to reaching WHO HCV elimination goals.

*Trial registration* NCT05401136.

## Background

The World Health Organization has set ambitious goals to eliminate hepatitis C virus (HCV) as a public health threat, aiming at a 90% reduction of new HCV infections and a 65% reduction of HCV related mortality [1, 2]. Disease progression models indicate only 24% of high-income countries are on track for achieving these goals before 2030 at the current rate of diagnosis and treatment [3, 4]. Expansion of efforts is therefore required.

One paramount strategy to reach HCV elimination goals is micro-elimination in specific target populations where HCV is prevalent, such as people who use drugs (PWUD). An estimated ~ 3,400 HCV RNA positive former and current injecting drug users remain in the Netherlands, despite availability of free HCV care across the country [5]. Injecting drug users are a subpopulation of PWUD and prevalence in the total Dutch PWUD population is currently unknown.

Studies indicate that HCV-infected PWUD may frequently get lost in the HCV care chain from diagnosis, to initiation of treatment and follow-up [6]. Recent studies showed that a median of 50% of PWUD with HCV visit the HCV specialist after testing positive and subsequently only 53% of those initiate treatment [7]. Loss to follow up during (median 2.5%) and after treatment (median 7.1%) were comparable to the general population [7]. Between 2013 and 2016 the Dutch HCV standard of care in addiction centers was evaluated and improved. Although major strides were made, linkage to hospital care and treatment remained as significant barriers due to DAA restrictions [8].

Over the past decade, DAA therapy became widely available and is now standard care globally. Recently, the use of pangenotypic DAAs, which bypass genotyping and simplify therapy choice, were recommended by both American Association for the Study of Liver Diseases (AASLD) and European Association for the Study of the Liver (EASL) [9, 10]. Furthermore, evaluation of liver fibrosis can be done using non-invasive, inexpensive and reliable biomarker panels, further reducing complexity of HCV care and burden for patients [10]. As a result, initiatives to offer decentralized HCV care for PWUD have been studied. A recent meta-analysis showed increasing linkage to care, with increasing levels of decentralization [11]. With full decentralization, HCV testing and treatment at the same site, showed the highest linkage to care of 72%, whereas without decentralization this was only 47%. Treatment uptake rate showed similar results [11]. Successful implementation of decentralized pathways are therefore the next step in achieving HCV elimination in PWUD [12, 13].

Previous studies have examined challenges in implementation of HCV care for PWUD outside of the hospital care setting [14–17]. Linkage to HCV care in PWUD may be reduced as a result of poor venous access due to damaging effects of intravenous drug use which precludes laboratory monitoring of treatment. A recent qualitative telephone interview study in general practitioners who prescribe opioid agonist therapy and addiction care specialists also identified logistic barriers like lack of testing on-site, and difficulty in liver fibrosis assessment [18]. There remains a need for addiction care focused research emphasizing residual local barriers, for instance stigmatization and lack of support for task shifting at management level. Nurses also play a vital role in addiction care, their perspectives are lacking in literature.

Dutch addiction care is provided through an integrated trajectory and joins government, municipality, justice system and health service providers with common goals of public order as well as public health and quality of care and treatment. Addiction care centers are a part of mental healthcare and deliver care that targets specific populations, provide outreach, night shelters, outpatient- and inpatient addiction treatment facilities. In addition, these institutions provide and coordinate reintegration projects, daytime activities, education and harm reduction (i.e. needle exchange programs) [19].

We aim to evaluate the effectiveness and feasibility of the HCV: Chain of Addiction Care project, which decentralizes HCV care in Dutch addiction care. This mixed-method study consists of (1) an observational study to explore its effectiveness in achieving 90% hepatitis C elimination, and (2) a qualitative study to explore facilitators and barriers for its implementation.

## Methods

### Study design

This mixed-method study consists of two parts. Part 1 is a multi-center observational cohort study that will study the effectiveness of the structured implementation of CAC in a PWUD population receiving addiction care. Part 2 is a qualitative study using semi-structured interviews regarding addiction care professionals’ personal perspective on barriers and facilitators for implementation of the CAC pathway. We will focus on addiction care specialists and nurses, as well as board members in addiction care as all play a vital role in successful implementation of decentralization projects. The interviews will either take place face-to-face or digitally using a videoconferencing platform. The interviews will be audio recorded and transcribed verbatim. The mixed-method design will be integrated into the RE-AIM framework to allow for evaluation using a well-established method for implementing community-based health interventions. The RE-AIM framework facilitates the translation of research to practice with attention for contextual factors, as opposed to only efficacy or effectiveness [20]. RE-AIM involves evaluation in five dimensions (Reach, Effectiveness, Adoption, Implementation, Maintenance), resulting in a more explicit description of the strategy. RE-AIM has been used extensively in over 450 publications on study planning or intervention evaluation [21].

### The chain of addiction care (CAC) project

**Fig. 1:**
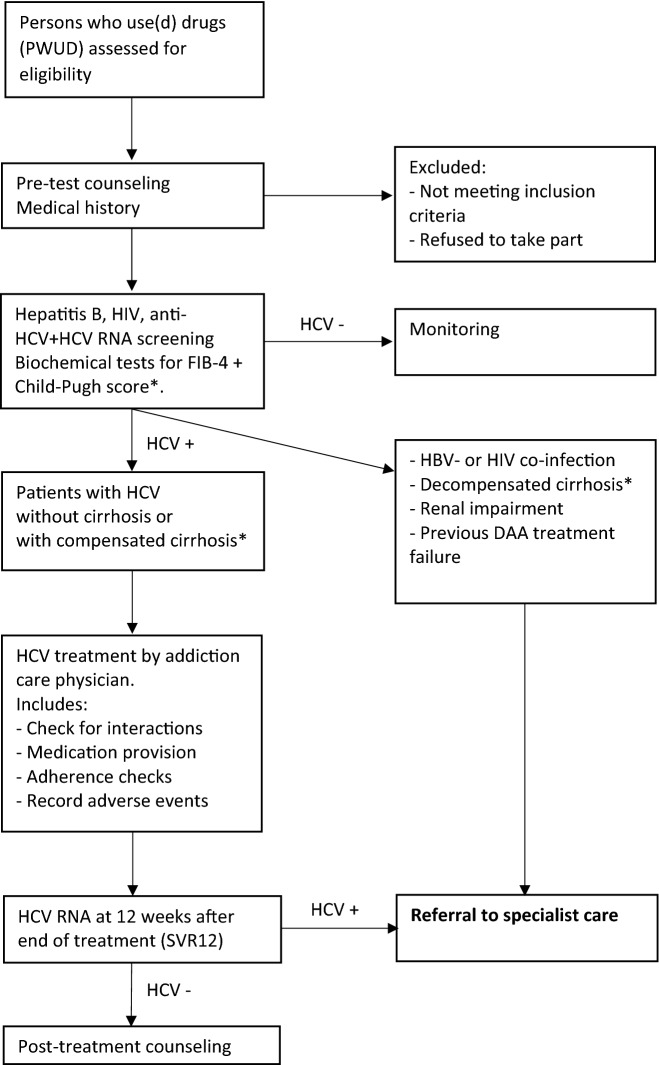
*CAC flowchart* *decompensated cirrhosis as indicated by FIB-4 score (> 3.25) and Child–Pugh score B or C; compensated cirrhosis as indicated by FIB-4 score (> 3.25) and Child–Pugh score A

The CAC pathway is a new approach to HCV care in the Dutch addiction care setting, in line with regular HCV care guidelines [9, 10]. All five addiction care organizations in the Nijmegen Institute for Scientist-Practitioners in Addiction (NISPA) collaboration will be invited to participate but we will also reach out to other major addiction care organizations to achieve nationwide coverage. All centers will be invited within a timeframe of two months. There will be no financial compensation for participation, however participation will include free education on hepatitis C. We anticipate that at least four out of five NISPA organizations and at least two non-NISPA organizations will participate. We will focus on centers that provide outpatient care, outreaching care or methadone posts, as these settings will contain most patients at risk for bloodborne infections and be suitable to provide hepatitis C care. Unfortunately, inpatient hepatitis C treatment is not currently reimbursed in the Netherlands. All phases of HCV care tasks are shifted to the addiction care physicians (ACP) and nurses, from case finding in patients with a risk for bloodborne infections to counselling, testing, treatment and follow-up. Patients at risk who visit the participating centers will be educated on viral hepatitis and HIV and motivated to undergo venipuncture. In the majority of patients, pangenotypic DAA treatment will be provided by the ACP after evaluation of biomarker panels (Fig. 1).

CAC is a project that is part of the portfolio that is offered by HepNed, a collaboration between hepatologists and infectious disease physicians. HepNed coordinated the nationwide hepatitis C retrieval program CELINE [22]. During the CAC project, as addiction care teams are familiarized with HCV care, a HepNed expert is available for advice and instruction. In some cases treatment provided by a viral hepatitis expert is preferred: in patients with co-infection hepatitis B or HIV, renal impairment (eGFR < 30 ml/min/1.73m^2^) and in patients who previously failed DAA therapy or patients who present with decompensated cirrhosis (Child–Pugh B and C). In case of advanced fibrosis or compensated cirrhosis (Fib-4 > 3,25 and Child–Pugh A), patients receive hepatitis C treatment in addiction care and are simultaneously referred for fibrosis analysis and cirrhosis follow-up in secondary care. Patients who fail treatment will be referred to specialist care. After completing the treatment, patients will be counselled and reintroduced into established health screening programs. Patients with an HCV reinfection will be treated as new patients and can be treated on-site again.

Implementation requires a dynamic and tailored approach to the local situation in the individual addiction care center based on variation in availability of on-site venipuncture, outreaching healthcare providers and existing collaborations with viral hepatitis experts. Derived from barriers found in previous studies, CAC interventions include DAA provision in methadone distribution posts and a limit to excess blood withdrawals by testing in combination with health screenings and implementation of reflex HCV-RNA testing.

### Study population

In Part 1 addiction care physicians will invite consecutive people who inject(ed) drugs or have other risk factors for HCV infection and who visit the CAC addiction care centers to participate. In order to be eligible for this study, a subject must be aged 18 years or older and able and willing to give informed consent. For the duration of the project, as many patients as possible will be included. There are no exclusion criteria for this study. Patients will be followed-up until the end of their HCV care journey. Since the CAC pathway is standard care in a decentralized setting, no comparison group can be formed. In part 2 we will invite addiction care physicians, nurses and addiction care policy makers employed both in centers participating in CAC and centers who do not.

### Study outcomes

The primary outcome in part 1 is HCV viral elimination, defined as percentage of patients that achieved SVR among identified HCV RNA positive patients. Secondary outcomes are total number of patients tested, prevalence of HCV antibody and RNA positivity among tested patients, fibrosis stage in HCV positive patients as found by FIB-4 and/or Child–Pugh scores, decentralized treatment initiation rate, treatment completion, sustained virologic response rate and referral. The main study outcomes for part 2 are the barriers and facilitators contributing to (un)successful implementation of the HCV care pathway for PWUD in the Netherlands.

### Data collection and management

The main study parameters collected are: acceptance rate of (on-site) testing, HCV prevalence, treatment acceptance rate, sustained virologic response (SVR) rates and re-infection rates. Furthermore, we will collect data on patient characteristics such as sex, age, history of drug use, medical history and comorbidities (including previous HCV infection and treatment), concomitant medication and available HCV-related clinical data (e.g. other risk factors, blood test results, liver fibrosis stage). All these parameters are stored in the electronic patient files by the patient’s treatment team as part of standard care. Data will then be collected anonymously by a member of the research staff according to the medical code of conduct [23]. A validated and GCP compliant data management program, CastorEDC, will be used for central data storage.

Interview recordings in part 2 of the study will be anonymized and used solely by the researches. The interview guide will address the following topics: preferred role of addiction center in HCV care: specifically for diagnosis, treatment and follow-up; experience and viewpoint on fully decentralized HCV care; real and anticipated barriers and facilitators for successful and sustainable implementation. Interviewee data collected includes participant characteristics such as sex, age, occupation and years of experience.

### Analysis

For the quantative study (part 1) we will use descriptive statistics. Primary and secondary endpoints, as well as baseline patient characteristics will be presented as percentages, means (± SD) or medians (IQR), depending on the variable and its distribution. No sample size is recorded as this is an observational prospective cohort study without a comparison group. All analyses will be performed using the statistical package SPSS, version 25.0 (SPSS Inc. Chicago, IL, USA).

In the qualitative study (part 2), Braun and Clarke’s approach to thematic analysis will be used to identify common themes across the interview transcripts [24]. The analysis will involve a six-phase process: familiarization with the transcripts, coding, searching for themes, reviewing themes, defining and naming themes and writing. The coding process will be data driven, in which codification is performed following an inductive method and themes are constructed from the data. Each transcript will be coded in pairs of 2 researchers. Qualitative thematic analysis will be performed using ATLAS.ti v9.1.6.

### Ethics

The Research Ethics Committee Arnhem-Nijmegen has waived formal ethical approval for this study, indicating it is not included in the Medical Research Involving Human Subjects Act. Eligible patients will be informed about the study by a member of the treatment team in the standard care pathway. While regular care continues as planned, patients are given sufficient time to consider participation in the study. Informed consent will be obtained before the collection of data by the coordinating researchers. Patients will be followed-up until the end of this study or until they withdraw consent. Due to the observational character of this study, participation poses no burden or risk for the participants. Participation leads to evaluation and may lead to improvement of the hepatitis care in addiction care facilities in the future, which benefits patients. Patients who decline participation in this study will still be offered access to onsite HCV care if the addiction care team can provide it.

## Discussion

HCV viral elimination in line with WHO goals is within reach [4]. Micro-elimination efforts in target populations like PWUD are essential in countries with a low HCV prevalence in the general population like the Netherlands. This study aims to test a decentralization model tailored to population and system needs, aimed to integrate HCV care within addiction care, with the specific objectives to (1) test effectiveness in achieving 90% elimination viral elimination rates; and (2) explore facilitators and barriers for implementation of decentralized HCV care within addiction care.

Efficient, low-threshold treatment for HCV-infected PWUD is one strategy of HCV-micro-elimination to reduce the human reservoir of HCV and contribute to HCV elimination goals of the WHO [25]. The findings of this study will strengthen the evidence base for decentralized HCV-care within addiction care. Furthermore, identification of facilitators and barriers for implementation of a decentralized HCV-care model within addiction care can identify best practices and feed into a blueprint to support scaling-up of HCV care decentralization. Thus, the current study will contribute to achieving WHO HCV elimination goals.

We make use of the RE-AIM framework to systematically evaluate all necessary dimensions for successful implementation of decentralized HCV care. It has to be acknowledged that commonly not all dimensions of the RE-AIM framework are covered within a single study. This also applies to the current study, where research objective one provides information on both reach and effectiveness of the intervention in real life, and the qualitative research objective two will mainly provide information on implementation (see Table 1). If successful, future studies should explore maintenance of decentralized HCV care within addiction care.

**Table 1:** Methods and endpoints in the RE-AIM framework

Quantative study endpoints: HCV care pathway (part 1)	RE-AIM dimension^a^	Qualitative study endpoints: barriers and facilitators (part 2)
• Demographics of population • Proportion of population tested	*Reach*	• Individual reasons for not participating • Recruitment of patients • Willingness to participate
• Viral elimination rate	*Effectiveness*	**–**
• Proportion of centers participating	*Adoption*	• Willingness to initiate the program
• Secondary HCV care endpoints (treatment uptake, completion) • Referral to hospital • Loss to follow up • Adherence	*Implementation*	• Strategies to improve implementation

^a^Maintenance is beyond the scope of this study but will be the focus of future studies

This study has multiple strengths. First, its nationwide implementation in different addiction care settings will ensure a heterogenic PWUD study population, increasing generalizability of findings. This allows us to translate our results to other addiction care facilities both in the Netherlands and other countries. Second, the mixed method design of this study allows for observational outcomes to be substantiated by qualitative findings that provide more in-depth information and understanding of the quantitative observations. Lastly, CAC efforts are guided by an experienced interdisciplinary team of behavioral scientists, addiction care experts, and viral hepatitis experts through HepNed. This will grant a unique opportunity to bridge the gap between addiction care and hospital care.

A limitation of this study is the lack of a control group, as the CAC pathway utilizes standard of care treatment. Also, we believe it would be unethical to deny patients in a control group safe and effective decentralized treatment, given the current evidence for decentralization of HCV care. Another limitation is that the ‘maintenance’ dimension of the RE-AIM model is beyond the scope of the study. The timeframe for this study does not include long-term follow-up. However, we do aim to establish low threshold communication between local hospitals and participating addiction care centers to fully integrate decentralized HCV care in the long-term. We therefore suggest to evaluate the long-term effect and adherence to implemented decentralized care models in future studies. Finally, it is important to note that not all PWUD seek care. In a field work study conducted in 2013, of 401 problematic opioid users (regular use with either criminal activity, comorbid psychiatry, or unstable lifestyle) 79.2% reported to have received addiction care in 2012. Based on this finding they estimated the size of the problematic opioid user population in the Netherlands at 14,000, a decline of 21% compared to 2009 [26]. Trends of problematic opioid use in addiction care in the Netherlands has since declined further, also their average age continues to rise, which indicates a minimal influx of new patients [27]. This means that there is a treatment gap, potentially hindering the reach of our intervention. However, given these findings our intervention within addiction care would still reach the majority of our target population.

To summarize, we will describe the nationwide implementation of CAC, a decentralized HCV care model for PWUD in the Netherlands. The study is aligned along the RE-AIM framework, aiming to contribute to HCV elimination among PWUD. Using a mixed methods approach, this study will substantiate current evidence for fully decentralized HCV-care, integrated in addiction care, and provide insights in potential facilitators and barriers for implementation and scaling-up of this decentralized HCV-treatment approach. This will contribute to achieving WHO hepatitis C elimination goals worldwide.

## Data Availability

Not applicable.

## Abbreviations

ACP: Addiction care physician
HBV: Hepatitis B virus
HCV: Hepatitis C virus
HIV: Human immunodeficiency virus
CAC: Hepatitis
C: Chain of addiction care project
DAA: Direct acting antiviral
IDU: Injection drug use
METC: Medical research ethics committee (MREC); in Dutch: medisch-ethische toetsings commissie (METC)
NISPA: Nijmegen institute of science-practitioners in addiction
OST: Opioid substitution therapy
PWUD: People who use(d) drugs
SD: Standard deviation
SVR: Sustained virological response
WHO: World Health Organization

## References

[1] WHO (2016). Combating hepatitis B and C to reach elimination by 2030.

[2] Spearman CW (2019). Hepatitis C. The Lancet.

[3] Gamkrelidze I (2021). Progress towards hepatitis C virus elimination in high-income countries: an updated analysis. Liver Int.

[4] van Dijk M (2021). The Netherlands is on track to meet the World health organization hepatitis C elimination targets by 2030. J Clin Med.

[5] Koopsen J (2019). Chronic hepatitis B and C infections in the Netherlands: estimated prevalence in risk groups and the general population. Epidemiol Infect.

[6] Grebely J (2013). Breaking down the barriers to hepatitis C virus (HCV) treatment among individuals with HCV/HIV coinfection: action required at the system, provider, and patient levels. J Infect Dis.

[7] van Dijk M, Drenth JPH, HepNed study (2020). Loss to follow-up in the hepatitis C care cascade: a substantial problem but opportunity for micro-elimination. J Viral Hepat.

[8] Kracht PAM (2019). Introducing hepatitis C virus healthcare pathways in addiction care in the Netherlands with a breakthrough project: a mixed method study. Harm Reduct J.

[9] Ghany MG, Morgan TR, AIHCG Panel (2020). Hepatitis C guidance 2019 update: American association for the study of liver diseases-infectious diseases society of America recommendations for testing, managing, and treating hepatitis C virus infection. Hepatology.

[10] European Association for the study of the Liver (2020). EASL recommendations on treatment of hepatitis C: Final update of the series. J Hepatol.

[11] Oru E (2021). Decentralisation, integration, and task-shifting in hepatitis C virus infection testing and treatment: a global systematic review and meta-analysis. Lancet Glob Health.

[12] Von den Hoff DW, Berden FAC, Schellekens AFA (2021). If the mountain won't come to Mohammed, then Mohammed must go to the mountain: Decentralisation of hepatitis C care for people who use drugs. United European Gastroenterol J.

[13] Mangia A (2021). Increased Hepatitis C virus screening, diagnosis and linkage to care rates among people who use drugs through a patient-centered program from Italy. United Eur Gastroenterol J.

[14] Assoumou SA (2021). Patients at a drug detoxification center share perspectives on how to increase hepatitis C treatment uptake: a qualitative study. Drug Alcohol Depend.

[15] Litwin AH (2019). Perceived barriers related to testing, management and treatment of HCV infection among physicians prescribing opioid agonist therapy: the C-SCOPE study. J Viral Hepat.

[16] Phillips C (2020). Improving access to care for people who inject drugs: qualitative evaluation of project ITTREAT-an integrated community hepatitis C service. J Viral Hepat.

[17] Tsui JI (2021). 'Treat my whole person, not just my condition': qualitative explorations of hepatitis C care delivery preferences among people who inject drugs. Addict Sci Clin Pract.

[18] Marshall AD (2020). Barriers and facilitators to engaging in hepatitis C management and DAA therapy among general practitioners and drug and alcohol specialists-The practitioner experience. Drug Alcohol Depend.

[19] Schatz E (2011). IDPC Briefing Paper The Dutch treatment and social support system for drug users. Int Drug Policy Consort.

[20] Sweet SN (2014). Operationalizing the RE-AIM framework to evaluate the impact of multi-sector partnerships. Implement Sci.

[21] Glasgow RE (2019). RE-aim planning and evaluation framework: adapting to new science and practice with a 20-year review. Front Public Health.

[22] Isfordink CJ (2022). Hepatitis C Elimination in the Netherlands (CELINE): how nationwide retrieval of lost to follow-up hepatitis C patients contributes to micro-elimination. Eur J Intern Med.

[23] Federation of Dutch Medical Scientific Societies; The code of conduct for the use of data in health research. 2004.

[24] Braun V, Clarke V (2006). Using thematic analysis in psychology. Qual Res Psychol.

[25] Organization WH (2017). Report WHO Global health sector strategy on viral hepatitis.

[26] Cruts G, V.L.M., Buster M. Aantal en kenmerken van problematische opiatengebruikers in Nederland. Utrecht/Amsterdam: Trimbos-instituut/GGD Amsterdam, 2013.

[27] Nationale Drug Monitor. Opiaten 5.6 Hulpvraag en incidenten—Nationale Drug Monitor. 2022; https://www.nationaledrugmonitor.nl/opiaten-hulpvraag-en-incidenten/. Accessed on 2 Nov 2022.

